# Supplementary material for: Correlative Multi-Modal Microscopy: A Novel Pipeline for Optimizing Fluorescence Microscopy Resolutions in Biological Applications
Source: Cells. 2023 Jan 17;12(3):354. doi: 10.3390/cells12030354 (PMC9913119; doi:10.3390/cells12030354)
Supplement: Supplementary file 1 [file cells-12-00354-s001.zip › cells-2099473-supplementary.pdf]

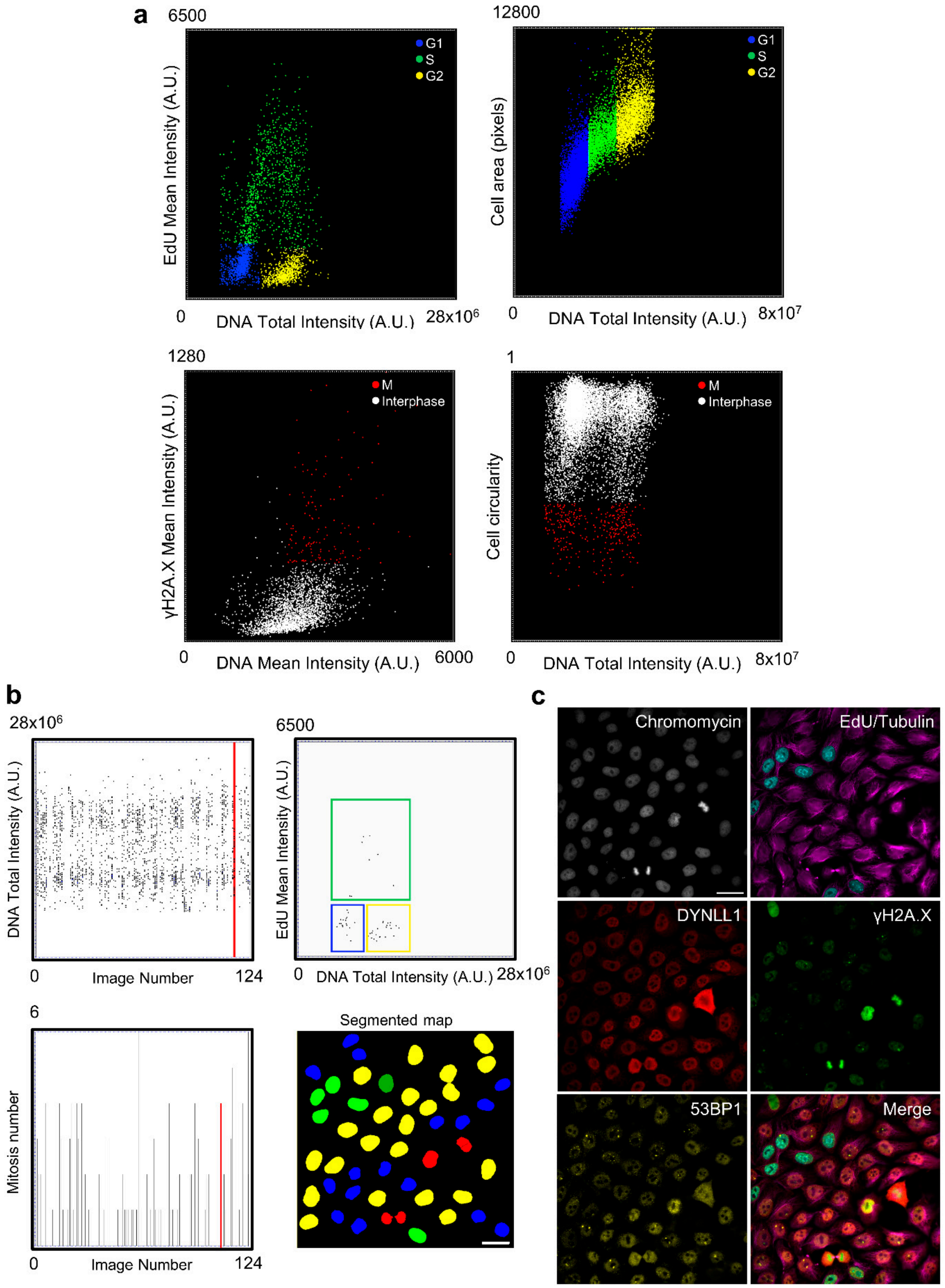

Figure S1. Representative A.M.I.CO analysis for the cell-cycle phases identification in exponentially growing MCF10A cells. **(a)** The dot-plots report the bivariate distribution of DNA content (X-axis) versus i) EdU mean intensity signal (left) or cell area (right) employed to detect the cell-cycle distribution: G1 (blue), S (green), and G2 (yellow). M cells (red) were identified based on DNA content together with γH2A.X mean intensity signal (indirect mitotic marker) and circularity (morphological

parameter). **(b)** Cell-cycle subpopulations (G1, S, G2, M) were selected by logical gates. Shown is the cell-cycle distribution reconstruction on a representative image: i) images can be retrieved from the data collection according to a file index corresponding to the order of image acquisition (Dot Plot: X-axis: Image Index; Y-axis: DNA total intensity); ii) bivariate analysis of cell-cycle phases (DNA versus EdU mean intensity) per selected image; iii) Mitotic cells were selected in the image according to the above mentioned procedure (Image Index versus mitosis number); iv) Color-coded segmentation map of the cell-cycle phases reconstruction in the target image. **(c)** Samples are stained for the detection of EdU, DynLL1, 53BP1, DynLL1, Tubulin, γH2A.X and DNA (Chromomycin). Scale bar: 30 μm.

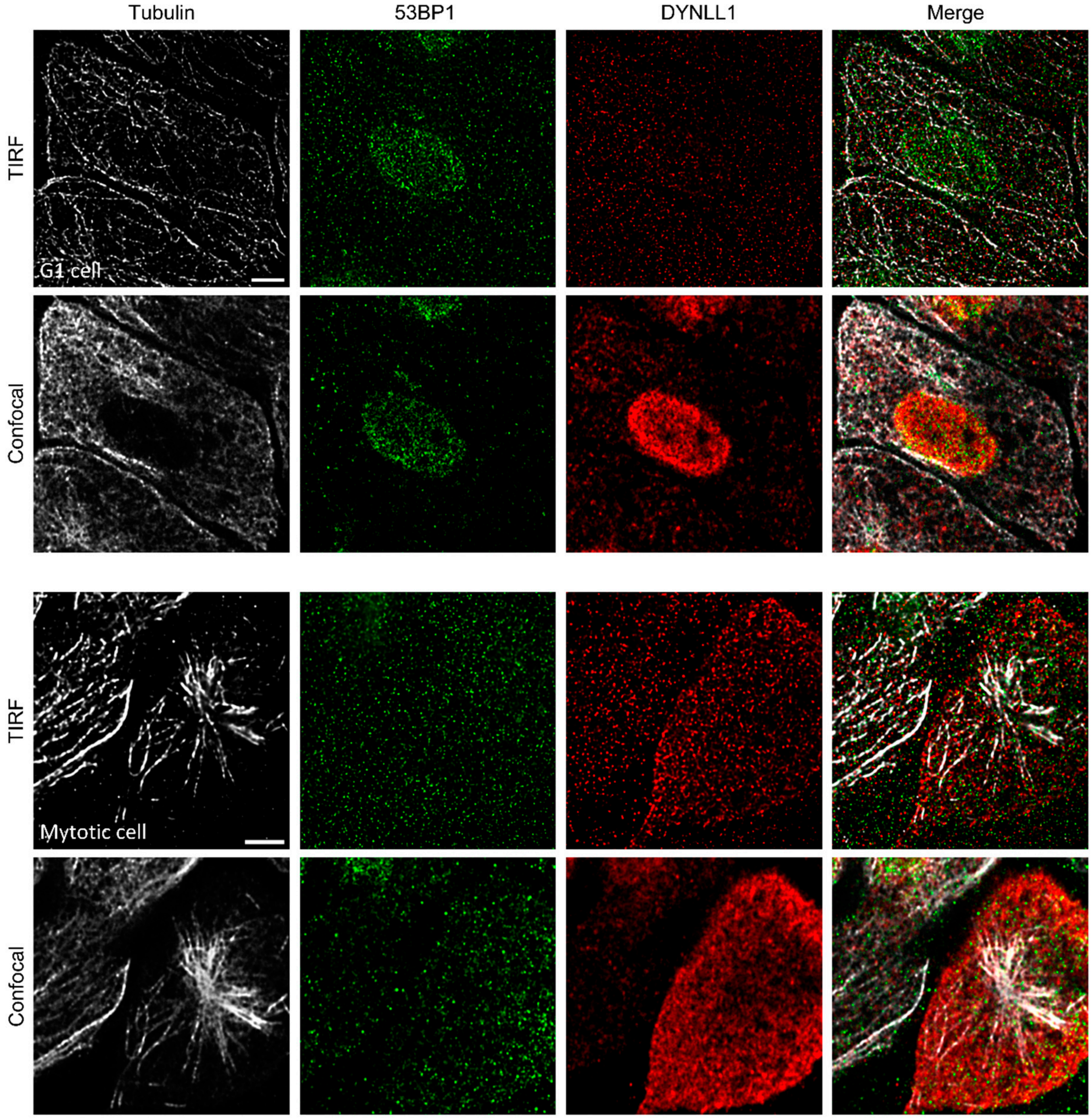

Figure S2. Comparison between TIRF and Confocal images of MCF10A basal membrane. Shown is the resolution improvement obtained by TIRF illumination on protein distribution of Tubulin, 53BP1 and DynLL1 at cellular basal plane. The images showed the same Z-plane and were employed for the image alignment in correlative reconstruction. Scale bar: 10 μm.

Supplementary Protocol. Detailed protocol of the correlative acquisition and registration.

Supplementary protocol:

Correlative dSTORM and confocal microscopy acquisition pipeline

Preliminary setup preparation

Timing: [varies]

1. Turn on the imaging setup.
2. Prepare fresh imaging medium (as described in Materials and Methods section).
3. Place a 125-µl drop of imaging medium onto the center of a 35mm Glass Bottom culture dish. Place one end on the coverslip on the dish and, with a tweezer, gently lower the coverslip onto the slide so that the medium covers the entire section under the coverslip without forming any bubbles.
4. Position the specimen on the microscope and focus on a ROI with the objective used for 2color-STORM imaging (100x NA1.49 in our case). After turning on the lasers at minimal power (or maximum power with a Neutral Density filter), the immunolabeling should be visible in the, red and far-red channels according to the performed staining.
5. Align the laser wavelengths. Set the TIRF illuminator incidence angle to the optimal signal to noise ratio (typically near the critical angle, which corresponds to TIRF illumination).
6. To check the blinking of the fluorophores, increase the power of excitation lasers, used for 2color-STORM, to maximum; the sample should bleach within seconds, and spontaneous blinking of fluorophores should be clearly visible. Upon brief pulses of illumination with low-power 405-nm laser, the density of blinking events should transiently increase.
   Note: the region of the sample used for the optimization will be bleached, and thus will be lost for quantitative imaging. Move to another region.

Step-by-step method details

Widefield Imaging

Timing: [5-10 min]

Here we describe the standard widefield imaging of the confocal targets, optimizing the widefield camera settings to the format of STORM acquisition module, combined with a 0.4X Relay lens.

7. Switch to the widefield module and focus on the ROI with the STORM objective used for imaging. After turning on the LED light sources at minimal power, the immunolabeling should be visible in the blue/green, red and far-red channels.
8. Select the channels of the confocal target. Typically, the blue and green bandwidths because red and far-red wavelength are selected for STORM dyes.
   Note: in our data, thanks to different immunostaining patterns, the blue dye (cytoplasm) did not interfere with STORM acquisition (nucleus).
9. Switch to the STORM module and adjust the ROI size (um x um) to the format of the CMOS image of STORM acquisition module, which is coupled with a 0.4X Relay lens. In this work, we applied a ROI of 620x620 pixel with 60nm of pixel size for widefield acquisition, which can be matched with 256x256 pixel ROI image with 0.4X Relay lens (160nm/pixel).
   IMPORTANT: if the STORM setup is not equipped with the relay lens, go directly to the confocal acquisition.
10. Optimize the microscopy parameters for widefield imaging. Select a long/short-pass dichroic mirror for the activation of the Perfect Focus System. Set the excitation powers, combined to the respective single-band filter cubes, to ensure optimal signal to noise ratio and minimal crosstalk and photobleaching. Select the most suitable exposure times (usually between 50 and 400 ms), based on the density pattern and the fluorescence intensity of the sample. Binning should be avoided.
11. After optimizing the imaging parameters and camera setting, acquire the widefield image (WF).

Confocal Imaging

Timing: [15 min]

Here we describe imaging of blue/green channels using a confocal system with a higher spatial resolution.

12. Switch to the confocal module. Select the 405nm and 488nm light paths.
13. Select the 405nm and 488nm lasers and check the profile of interest of the cell in the field of view, maintaining the focal position set during the widefield acquisition.
    CRITICAL: switching to confocal module, check if the long/short-pass dichroic mirror is on and check that the plane of focus is maintained.

14. Optimize the microscopy parameters for confocal imaging. Set bidirectional galvanometric (or resonant) scanning and adjust line average, pixel dwell time, laser power, bandpass filters, and gain to increase quality signal.
    Note: In this work we employed a Nikon A1R system. We set 5–20% of 405 (23.1 mW) and 488nm (79.1 mW) laser powers for confocal imaging with 450-490 and 510-530 bandpass filters, respectively. The emission filter is selected to minimize the crosstalk with the signal from other channels. The pinhole size is set to 0.8 Airy unit.

15. Avoid saturated pixels in the image. Use the same pixel size (60nm/pixel) of the widefield acquisition and a scan area similar to widefield format.
    CRITICAL: in galvanometric scanning the scan area ROIs are preselected. It is recommended to use an image format greater than widefield camera settings (e.g., 1024x1024 pixels).
16. Set acquisition parameters for 3D acquisition with the dedicated module. In NIS Elements software, open the Capture Z-Series module for 3D acquisition and select the Z-scanning parameters (Top/Bottom planes (volume range), step range and Z-Device).
    Note: The step range defines the number of steps in the selected volume. In our experiment, we fix the step range at 0.16 um.

17. Run the 3D confocal acquisition.

dSTORM Imaging

Timing: [20 min]

Here, we described the single-molecule acquisition procedure. 2-color STORM imaging may be carried out with the basic STORM module of NIS Elements software or with an automated acquisition pipeline (JOB), created with a dedicated module of the software.

18. Acquire the widefield image (WF STORM) of the molecular target of confocal image with 488nm excitation laser. Use the STORM camera settings which matched with the ROI size of the widefield acquisition module. (See Step. 11).
    CRITICAL: use the same focal plane of the first widefield acquisition.

19. Optimize the microscope parameters for STORM imaging for each channel. Turn on the imaging laser to a power of 70-90% and allow the fluorescent molecules in the sample to reach the dark state. Blinking should be visible at this stage. Set the camera parameters for optimal S/N ratio. Considering the density pattern of the staining and the duration of dye blinking events, it is generally suitable to use an exposure time of 20ms. Binning should be avoided.
20. Set the activation laser (405 nm) at minimal power and increase the power gradually until blinking becomes sufficiently dense. In our system, typically 1–3% of 405-nm

laser power for Alexa Fluor-647 (or DyLight650) STORM imaging and 5-10% for the Cy3 (or CF568) can be used to optimize blinking of the dyes.

21. Set the activation mode and the number of frames. For dSTORM, we employed continuous activation mode (both the activator and imaging lasers are continuously on). The optimal number of frames per image depends on the type of experiment. In this work, we selected 15.000 frames per channel in continuous mode.
22. Activate a channel for drift correction. In our experiments the green channel (488nm) is employed to excite nanodiamonds fluorescence (40nm-Fluorescent Nanodiamonds (FNDs) conjugated to Streptavidin, Adamas Nanotechnologies). Set the acquisition interval of 488nm-channel during STORM imaging; we collected FNDs signal with a frequency of 1 every 1000 frames during the acquisition of each channel.
    Important: In each channel, every acquisition starts with a transitory phase to push all the molecules to the dark state. The duration of the transition depends on the employed dye, density of labelled molecules and power of the excitation and activation lasers.
23. Run the 2color-STORM real time acquisition on the two channels sequentially. During acquisition, adjust the activation laser power (405 nm) to optimize the number of blinking events.

dSTORM Image reconstruction

Timing: [varies]

Here, we described the dSTORM image reconstruction of 2-color channels, aligned with 40nm-Fluorescent Nanodiamonds.

24. Open the software employed for detection of single molecule events (in our case NIS Elements Offline N-STORM Analysis module; several freeware software are available to perform molecule localization and reconstruction, such as ThunderSTORM, QuickPALM or RapidSTORM.) in STORM images.
25. Open the 647nm-channel STORM dataset and find the proper intensity parameters to identify single-molecule photon emissions
26. Select the minimum intensity to identify fluorescent Nanodiamonds peaks.
27. Activate the correction of the spatial drift between frames
28. Finally, run the analysis. Generally, a reconstructed STORM image and a molecule list in a binary format are generated.
    Note: For 2D STORM experiments with high labeling density, it might be useful to discriminate the single events by deconvolving overlapping Gaussian PSFs. The aim of the analysis of the images acquired in a STORM experiment is the identification of blinking events and fitting a Gaussian function on each single-molecule image to determine the centroid position.

IMPORTANT: When using FNDs their localizations from the corresponding channel (488 nm excitation) are employed as reference point to calculate the drift correction. However, FNDs are detected in all channels, given their spectroscopy properties. Localizations in their proximity should be consequently removed considering their physical size (40 nm) and the obtained localization precision.

29. Reconstruct the final STORM image by superimposing a Gaussian PSF to every localized molecule. The amplitude of the curve is proportional to the number of photons detected: the conversion factor (gray levels-photon number) is a parameter dependent on the employed camera generally provided by the producer. Choose the gaussian width (10 and 50nm in this work) of the single-localization PSFs and the format (10nm/pixel) of the reconstructed image.
30. Save the final STORM image
31. Repeat steps 14-20 for 561-channel STORM dataset.
32. Align the two Channels (647 nm and 561 nm) by the Nanodiamonds-peaks positions, calculated in the drift-correction channels.

Correlative Image reconstruction

Timing: [10min]

Here, we described the 3D-confocal and 2D-dSTORM images alignment to generate a correlative image.

33. Open the following images:
    i. Widefield image, acquired with widefield module (WF)
    ii. Confocal image
    iii. Widefield image, acquired with STORM module (WF STORM)
    iv. 2 reconstructed STORM images
31. Use a dedicated images alignment algorithm to align WF and confocal image. In this work we used the Multimodal Image Registration tool of NIS Elements software, based on the manual multi-point affine transformation (also implemented by TurboReg plugin in ImageJ software), which creates a new document with two perfectly aligned images.
32. Define which image is “fixed” (WF) and which one is “moving” (confocal) and finds reference points contained in both images. Once selected, the algorithm aligns the images perfectly (the two images had the same pixel size of 60nm/px).
33. Finally, crop the resulting document to the format of WF image.
34. Merge 2-channel STORM and WF STORM images. Since, STORM images and WF STORM image have been sequentially acquired with the same hardware (0.4X Relay lens CMOS camera), they resulted already aligned.
35. Then, align the resulting image to the just aligned document (WF+confocal).

Note: WF and WF STORM images had the same image format (see Step 9).

36. Finally, remove the widefield images and obtain a correlative image composed by multiple super- and high-resolved channels. The two STORM images were previously aligned each other, by using the Nanodiamonds-peaks positions (see Step 32).
    Note: widefield images were used as "surrogate images" for the correct alignment of confocal and dSTORM.
